# Current perception threshold testing in chronic ankle instability

**DOI:** 10.1186/s12891-021-04345-y

**Published:** 2021-05-18

**Authors:** Ran Zhang, Xi Zhang, Yaping Chen, Weiqun Song

**Affiliations:** 1 Department of Rehabilitation, Xuanwu Hospital, Capital Medical University, 45 Changchunjie, Beijing, 100054 China; 2 Department of Rehabilitation, Beijing Tongren Hospital, Capital Medical University, 1 Dongjiaominxiang, Beijing, 100730 China

**Keywords:** Chronic ankle instability, Current perception threshold, Sensory nerve function

## Abstract

**Background:**

Damage to sensory input is an underlying pathology of chronic ankle instability (CAI). Therefore, it is necessary to evaluate the sensory function of patients with CAI. The present study quantitatively evaluated sensory nerve function in patients with CAI and healthy controls using current perception threshold (CPT) measurements, as well as the influence of sex, age, and body mass index (BMI) on CPT values and the relations between CPT frequencies.

**Methods:**

Fifty-nine subjects with CAI and 30 healthy controls participated in this study. CPT values at the anterior talofibular ligament region were recorded on the injured and uninjured sides in CAI patients and on both sides in the healthy control group. Between group differences were compared. The influence of sex, age and BMI on CPT values was evaluated. Correlations between different frequencies were also studied.

**Results:**

There were no significant differences in age, sex, height, weight or BMI between the CAI and healthy control groups. The CPT values did not show a significant difference by sex. The CPT values did not significantly correlate with age or BMI. Compared to the control group, the CAI group had significantly higher CPT values on the injured and uninjured sides under 250-Hz and 5-Hz electrical stimuli; the difference between the groups was significant (*p* < 0.01), and the effect size were large. No significant difference was observed under 2000-Hz stimuli. There were correlations between CPT values at different frequencies (p < 0.01), especially 250 Hz and 5 Hz.

**Conclusion:**

The present study revealed increased sensory thresholds in 250-Hz- and 5-Hz-related sensory nerve fibres in the injured and uninjured ankles of patients with CAI. This increase may indicate dysfunction of A-delta and C fibres. Sex, age and BMI did not significantly impact CPT values. There were correlations between CPT values at different frequencies, especially 250 Hz and 5 Hz.

**Level of evidence:**

Level III, case-control study.

## Background

Ankle sprain is one of the most common injuries in daily life and sports. Up to 70% of individuals who sustain an acute ankle sprain may develop residual physical disability [1]. Perceived instability and repetitive ankle sprains are known as chronic ankle instability (CAI) [2]. The underlying neurophysiological mechanism of CAI is not clear. After the initial ankle sprain, damage to the ligaments and other tissues around the ankle triggers sensorimotor changes via inflammatory and pain mediators, resulting in sensory-perceptual and motor-behavioural impairments. Personal and environmental factors also influence clinical outcomes [3].

Postural control is dependent on integrated sensory input, central information processing in the central nervous system, and motor output [4]. Errors in the joint repositioning test are used in assessments of CAI-associated deficits in joint proprioception. An increase in errors in ankle joint repositioning was reported [5]. Cho et al. [6] used an isokinetic test and showed decreased joint position sensation in CAI participants. A systematic review reported that proprioception of the injured ankle in patients with CAI was impaired compared with that of the uninjured contralateral limb and in healthy people [7]. The superficial sensory test has been less studied in CAI. Navarro-Santana et al. [8] used an electronic algometer to measure the pressure pain threshold in CAI amateur male soccer players and found higher pressure pain sensitivity.

Sensory function may be evaluated subjectively and objectively [9]. Subjective evaluation is performed using physical examinations, e.g., the pinprick test and vibration sensory test. However, it is difficult to standardize and quantify these subjective tests. Quantitative objective measurements are comparable. The two-point discrimination test, thermal pain threshold test, filament-prick pain threshold test, pressure pain threshold test, and joint position sensory test provide quantitative measurements.

Current perception threshold (CPT) testing selectively evaluates the A-beta, A-delta, and C fibres in sensory nerves. A-beta fibres are large myelinated fibres that are activated by 2000-Hz electrical stimuli and transmit vibration and tactile messages. A-delta fibres are thinly myelinated fibres that are activated by 250-Hz electrical stimuli and transmit fast pain signals. C fibres are unmyelinated fibres that are activated by 5-Hz electrical stimuli and transmit slow pain signals [10, 11]. The Neurometer® CPT/C is a transcutaneous electrical stimulator that delivers sinusoidal electrical stimuli of 2000, 250, and 5 Hz via surface electrodes [12]. CPT testing may be performed on any cutaneous/mucosal area of the body and can be used for the evaluation of diabetic neuropathy [13, 14], diabetic foot problems [15], carpal tunnel syndrome [16], fracture [9], peripheral nerve injury [17], pain [18], restless leg syndrome [19] and other clinical disorders. High reliability has been confirmed across CPT testing applications [11, 18]. To the best of our knowledge, there have been no reports describing CPT testing in CAI patients.

Previous studies have shown inconsistent results regarding sex and age differences [18, 20, 21]. Uddin [18] studied CPT values in mechanical neck disorder participants and found no significant correlation of sex or age with CPT values. Chang [20] reported consistent results in pharyngeal paraesthesia patients. Conversely, Nakatani-Enomoto [21] studied healthy participants and found higher CPT values in men than in women and in older individuals than in younger subjects. Seno [22] studied healthy participants and reported higher CPT values in males than in females. The difference may be caused by differences in body fat percentage and body water percentage. Pitei [23] studied healthy and diabetic neuropathy participants and reported a stronger correlation between CPT values at 2000 Hz and 250 Hz and between CPT values at 250 Hz and 5 Hz than between CPT values at 2000 Hz and 5 Hz. These differences may reflect CPT neuroselectivity. Therefore, we examined differences according to sex and correlations between age, body mass index (BMI) and CPT values, as well as internal correlations between three frequencies, in CAI participants.

Therefore, in the present study, we quantitatively evaluated CPT values at the anterior talofibular ligament region using CPT measurements, compared CPT values according to sex, and calculated correlations between age, BMI and CPT values. We hypothesized that patients would exhibit higher CPT values due to sensory dysfunction than the controls. Sex, age, and BMI may influence CPT values. There may be internal correlations between different frequencies.

## Methods

This study was designed as a case-control study.

### Patients

Fifty-nine subjects with CAI (male: 10, female: 49) and 30 healthy controls (male: 8, female: 22) were recruited from a regional hospital from April 2017 to November 2018. Healthy controls were staff and medical students in the hospital. The inclusion and exclusion criteria for patients with CAI were selected according to criteria from the International Ankle Consortium [24]. The inclusion criteria for the CAI group were (1) at least 1 significant lateral ankle sprain resulting in inflammatory symptoms and 1 or more days of activity loss. The initial sprain occurred at least 12 months prior to the study. The most recent injury occurred at least 3 months prior to the study. (2) A continuing feeling of “giving way” and/or recurrent sprain and/or “instability”. Individuals in the healthy control group had never experienced an ankle sprain or episode of instability. The exclusion criteria for both groups were (1) previous lower limb surgery, fracture or acute injuries to the musculoskeletal structures, (2) neurological disorders or other systemic diseases, such as diabetes mellitus, that impact foot and ankle sensation and/or function, and (3) generalized joint laxity evaluated using the Beighton score [25].

This study was performed in accordance with the Declaration of Helsinki. Participants read and signed an informed consent form, which was approved by the ethics committee of our hospital, prior to the study.

### CPT measurement

CPT measurements were performed using a neurometer (Neurotron, Baltimore, MD, USA). A CPT value of 1 is equal to an output intensity of 0.01 mA with an available maximum of 9.99 mA. The stimuli were applied to the skin of the anterior talofibular ligament region (superficial peroneal nerve, L5 dermatome) of the injured and uninjured ankles in participants with CAI and both ankles in control participants by a well-trained doctor with experience in CPT testing administration. The order of the CPT testing in CAI participants and controls was randomly assigned using random number software. Values in the CAI group were recorded on the injured side and the uninjured side. Values in the control group were recorded on the random side and the control side, which were randomly assigned by the software, since the injured side may be the left or right side. The electrical stimuli were delivered via two 10-mm-diameter gold-plated electrodes at frequencies of 2000 Hz, 250 Hz and 5 Hz to the tested site. A doctor, who was blinded to participants’ group assignment, manually increased the electrical stimuli slowly until the participants reported a sensation of stimulation. The current was rapidly decreased and increased until the same threshold measurement was obtained on at least three consecutive trials [10]. The CPT was defined as the minimum electrical stimulus sensed by the participant [14]. To prevent guessing, “sham” stimulation was randomly delivered. When the tester pressed the sham stimulation button, the device did not send a stimulus. The tester then asked the participant if there was a sensation of current. If only true stimuli were reported by the participant, the test results were considered reliable. If the participant reported sham stimuli as true, the thresholds were retested. The duration of each stimulus was 3 s, with a between-stimuli rest of 5 s or longer until the residual sensation dissipated. Each trial lasted approximately 5 min, with 2 min of rest between each trial. The experiments were performed in a quiet room in the hospital.

### Statistical analysis

Statistical analyses were performed using SPSS 26.0 (IBM Co., Inc., Chicago, IL, USA). The normality of the variable distribution was evaluated using the Kolmogorov-Smirnov test. Continuous data are expressed as the mean ± standard deviation (SD). Independent t-tests were used to compare demographic data between the two groups, except by sex. The chi-squared test was used to compare sex differences between the CAI and control groups. Comparisons of CPT values between CAI patients and the control group were performed by 2 × 2 (group x side) repeated-measures ANOVA, and effect sizes (η^2^) were calculated, with 0.01 indicating a small effect size, 0.06 indicating a medium effect size and 0.14 indicating a large effect size [26, 27]. CPT values were compared between male and female patients with CAI by independent t-tests. Pearson correlations was used for correlation between age, BMI and CPT values and correlations between CPT values at the three different frequencies. Statistical significance was defined as *p* < 0.05.

## Results

### Demographic data

No significant between-group differences were present for age, sex, height, weight or BMI (Table 1).

**Table 1:** Participant demographics

	CAI (*n* = 59)	Control (n = 30)	p
Age, y	31.68 ± 10.36	29.70 ± 9.11	0.378
Sex	Male: 10, Female: 49	Male: 8, Female: 22	0.403
Height, m	1.64 ± 0.08	1.67 ± 0.08	0.060
Weight, kg	60.91 ± 13.34	66.93 ± 17.95	0.252
BMI, kg/m^2^	22.43 ± 3.54	23.59 ± 4.81	0.199
Time since last ankle sprain, m	12.75 ± 16.31		

*CAI* Chronic ankle instability, *BMI* body mass index

### Comparisons of CPT values between groups

CPT values are reported in Table 2. Significant between-group differences existed for CPT values at 250 Hz and 5 Hz. The injured and uninjured sides of patients in the CAI group showed higher CPT values than the random and control sides of the healthy controls (*p* < 0.01); the eta squared values were 0.176 (250 Hz) and 0.266 (5 Hz), which were large. No significant differences were found between the injured and uninjured sides of patients in the CAI group.

**Table 2:** Comparison of CPT (mA*100) values of the anterior talofibular ligament region

	CAI (*n* = 59)		Control (*n* = 30)		p	η^2^
	Injured side	Uninjured side	Random side	Control side		
2000 Hz	194.80 ± 64.58	217.81 ± 92.19	222.40 ± 49.31	222.93 ± 46.41	0.231	0.016
250 Hz	42.83 ± 28.49	38.62 ± 19.98	24.32 ± 9.28	25.68 ± 9.10	< 0.01	0.167
5 Hz	23.43 ± 18.53	18.84 ± 14.21	6.87 ± 1.56	7.64 ± 1.49	< 0.01	0.266

*CPT* Current perception threshold

### Comparison of CPT values between male and female patients with CAI

No significant differences were found between male and female patients with CAI (Table 3).

**Table 3:** Comparison of CPT (mA*100) values between the sexes

	Male (*n* = 10)	Female (*n* = 49)	p
2000 Hz	221.70 ± 82.47	189.31 ± 59.85	0.150
250 Hz	38.10 ± 25.38	43.80 ± 29.23	0.569
5 Hz	24.63 ± 21.60	23.19 ± 18.08	0.825

*CPT* Current perception threshold

### Correlations between age, BMI and CPT values

There was no significant correlation between the CPT value and age. No significant correlation between the CPT value and BMI was observed (Table 4).

**Table 4:** Correlation of influencing factors

	2000 Hz		250 Hz		5 Hz	
	r	p	r	p	r	p
Age	0.216	0.101	0.194	0.142	0.192	0.146
BMI	−0.198	0.133	−0.200	0.129	−0.153	0.248

*BMI* body mass index

### Correlation between CPT values at different frequencies

There were significant correlations between CPT values at different frequencies. The correlation between 250 Hz and 5 Hz was stronger than that between 2000 Hz and 250 Hz and that between 2000 Hz and 5 Hz (Table 5).

**Table 5:** Correlations of CPT values at different frequencies

	r	p
2000 Hz–250 Hz	0.419	< 0.01
2000 Hz–5 Hz	0.459	< 0.01
250 Hz–5 Hz	0.834	< 0.01

## Discussion

The pathogenesis of CAI is very complicated. We tested the CPT values in the anterior talofibular ligament area of injured and uninjured ankles. The results showed that patients with CAI had higher CPT values under 250-Hz and 5-Hz electrical stimuli than healthy controls, which were related to fast pain-related small myelinated A-delta and slow pain-related unmyelinated C fibres of the sensory nerves, respectively.

The injured ankle showed a trend of higher CPT values than the uninjured ankle in the CAI group under 250-Hz and 5-Hz stimuli. Both ankles in the CAI group showed higher CPT values than those in the control group. These differences may indicate inherent differences or changes after injury via chronic pain. Decreased sensitivity contralateral to sites of peripheral nerve injury has been reported and might result from peripheral lesions involving afferent fibres that alter central processing and reorganization at the spinal, subcortical, or cortical level [28]. According to the diffuse noxious inhibitory control theory, after heterotopic noxious stimulation, there is inhibitory modulation of pain pathways, including of the subnucleus reticularis dorsalis and its descending projections to wide-dynamic-range neurons [29].

Sensory disorder is one of the pathophysiological changes in CAI. Sensory function becomes impaired due to injury to the receptors in the ligaments, capsule, and tendons of the ankle [2]. Receptors include mechanoreceptors and nociceptors. Mechanoreceptors receive tactile, pressure, vibration, and motor information. Nociceptors received thermal and pinprick information.

Previous researchers have primarily reported on proprioception deficits in CAI [5–7] and less on superficial sensory disorders [8]. The CPT tests directly stimulated and tested the different subgroups of sensory nerve fibres. The Neurometer® CPT/C stimulated the three types of sensory nerve fibres: 2000 Hz activated large myelinated A-beta fibres; 250 Hz activated small myelinated A-delta fibres; and 5 Hz activated unmyelinated C fibres. Therefore, quantitative evaluations of different sensory fibres are available. Similar to our findings, Griffioen et al. [30] reported higher CPT values using 2000-Hz and 250-Hz stimuli in patients with chronic pain following lower extremity fracture. They also reported higher CPT values after lower body injury. The differences in frequencies may occur because not all sensory fibres are affected equally at the time of injury. Liao et al. [31] measured CPT values in trigeminal neuralgia patients and found that CPT values under a 5-Hz stimulus were decreased in the acute stage but increased in the chronic stage. The CAI patients in our study showed increased CPT values under 250-Hz and 5-Hz stimuli, which were related to the dysfunction of small fibres that conduct fast and slow pain signals.

Notably, the injured and uninjured sides showed increased CPT values under 250-Hz and 5-Hz stimuli in our study, and these bilateral changes may reflect inherent differences or changes after injury via central modulation. Previous studies have reported bilateral changes after unilateral ankle sprain and CAI [7, 23]. Sousa et al. [32] reported bilateral deficits in proprioception in the injured and uninjured limbs of CAI patients. Terada et al. [33] used transcranial magnetic stimulation to evaluate corticospinal excitability and reported a potential increase in corticospinal inhibition in CAI. Rosen et al. [34] used functional near-infrared spectroscopy to study cortical activation and found larger cortical activation variability in CAI. A systematic review reported decreased neural excitability of the soleus and the fibularis longus in CAI patients using the spinal H-reflex [35]. Further studies are needed regarding bilateral changes after unilateral ankle injury.

Previous studies have shown inconsistent results regarding the effects of sex, age and BMI. Uddin et al. [18] reported that age and sex were not correlated with CPT values. Nakatani-Enomoto et al. [21] reported higher CPT values in men than women and in older individuals than younger subjects. The present study found no correlations between sex, age or BMI and CPT values at any frequency. These inconsistencies may be due to the different sample sizes and objectives of the studies.

Our study revealed correlations between CPT values at different frequencies. Participants with higher CPT values at one frequency showed trends of higher CPT values at other frequencies. This result may be due to individual variability. The correlations may reflect the consistency of the sensory threshold test using CPT measurements. The correlation between 250 Hz and 5 Hz was stronger than that between other frequencies, which may be because the 250-Hz and 5-Hz stimuli activated small fibres, while the 2000-Hz stimuli activated large fibres. These may reflect CPT neuroselectivity. The findings are consistent with those reported by Pitei [23].

The results of the present study support the use of CPT testing as a quantitative sensory detection method for CAI. Further studies are needed to evaluate the recovery and prognosis values of this method.

However, there are some disadvantages to CPT tests. These testing procedures require participant reaction and feedback, similar to the hearing test. Therefore, they are not entirely objective methods. Nerve conduction velocity as measured using electromyography is an objective measurement, but there were reports of lower motor nerve conduction velocity [36], only sensory nerve studies. The conduction velocity reflects the function of large myelinated fibres [37].

There are some limitations to this study. First, this report was a case-control study performed at a tertiary referral centre, and unknown selection bias may have affected the study results. Second, only CPT values at the anterior talofibular ligament region were measured, and future studies are needed to investigate the CPT values at more sites in the lower extremities in CAI subjects. Third, participants in the control group were staff and medical students. Further studies may include a hypermobile group of patients who do not suffer CAI. Studies of the relationships between CPT tests and other sensory tests are also needed.

## Conclusion

The present study revealed increased sensory thresholds in the injured and uninjured limbs of 250-Hz- and 5-Hz-related sensory nerve fibres in patients with CAI. These results suggest dysfunction of A-delta and C fibres. Bilateral changes may be caused by inherent differences or neuromodulation. Sex, age and BMI did not significantly impact CPT values. There were correlations between CPT values at different frequencies, especially 250 Hz and 5 Hz. CPT testing has potential as a tool to quantitatively assess the function of sensory nerve fibres in CAI and monitor dynamic changes after treatment.

## Data Availability

The datasets during and/or analysed in the current study are available from the corresponding author upon reasonable request.

## Abbreviations

CAI: Chronic ankle instability
CPT: Current perception threshold
BMI: Body mass index

## References

[1] Herzog MM, Kerr ZY, Marshall SW, Wikstrom EA (2019). Epidemiology of ankle sprains and chronic ankle instability. J Athl Train.

[2] Galhoum AE, Wiewiorski M, Valderrabano V (2017). Ankle instability: anatomy, mechanics, management and sequelae. Sport Orthop Traumatol.

[3] Hertel J, Corbett RO. An updated model of chronic ankle instability. J Athl Train. 2019;54:572–88. 10.4085/1062-6050-344-18 PMC6602403 31162943

[4] Wikstrom EA, Naik S, Lodha N, Cauraugh JH. Bilateral balance impairments after lateral ankle trauma: A systematic review and meta-analysis. Gait Posture. 2010;31:407–14. 10.1016/j.gaitpost.2010.02.004. 10.1016/j.gaitpost.2010.02.004 20303759

[5] Arnold BL, De La Motte S, Linens S, Ross SE (2009). Ankle instability is associated with balance impairments: a meta-analysis. Med Sci Sports Exerc.

[6] Cho BK, Park JK (2019). Correlation between joint-position sense, Peroneal strength, postural control, and functional performance ability in patients with chronic lateral ankle instability. Foot Ankle Int.

[7] Xue X, Ma T, Li Q, Song Y, Hua Y (2020). Chronic ankle instability is associated with proprioception deficits: a systematic review with meta-analysis. J Sport Heal Sci.

[8] Navarro-Santana MJ, Albert-Lucena D, Gómez-Chiguano GF, Plaza-Manzano G, Fernández-de-las-Peñas C, Cleland J, Pérez-Silvestre Á, Asín-Izquierdo I (2019). Pressure pain sensitivity over nerve trunk areas and physical performance in amateur male soccer players with and without chronic ankle instability. Phys Ther Sport.

[9] Oh D, Yun T, Kim J, Choi J, Jeong W, Chu H, Lee S (2016). The measurement of the sensory recovery period in zygoma and blow-out fractures with neurometer current perception threshold. Arch Plast Surg.

[10] Nishimura A, Ogura T, Hase H, Makinodan A, Hojo T, Katsumi Y, Yagi K, Mikami Y, Kubo T (2004). A correlative electrophysiologic study of nerve fiber involvement in carpal tunnel syndrome using current perception thresholds. Clin Neurophysiol.

[11] Furuse N, Kimoto S, Nakashima Y, Ogawa T, Furokawa S, Okubo M, Yamaguchi H, Kawai Y (2019). Verification of the reliability of current perception threshold and pain threshold testing by application of an electrical current stimulus to mandibular mucosa in young adults. J Oral Rehabil.

[12] Medicine AA of E. Guidelines in electrodiagnostic medicine. Technology review: the Neurometer Current Perception Threshold (CPT). Muscle Nerve. 1999:523–31. 16921640

[13] Lv SL, Fang C, Hu J, Huang Y, Yang B, Zou R (2015). Assessment of peripheral neuropathy using measurement of the current perception threshold with the Neurometer ® in patients with type 1 diabetes mellitus. Diabetes Res Clin Pract.

[14] Masson EA, Boulton AJM (1991). The Neurometer: Validation and Comparison with Conventional Tests for Diabetic Neuropathy. Diabet Med.

[15] Nather A, Lin WK, Aziz Z, Ong CHJ, Feng BMC, Lin CB (2011). Assessment of sensory neuropathy in patients with diabetic foot problems. Diabet Foot Ankle.

[16] Kang EK, Lim JY, Shin HI, Gong HS, Oh JH, Paik NJ (2008). Comparison between nerve conduction studies and current perception threshold test in carpal tunnel syndrome. Neurophysiol Clin.

[17] Ziccardi VB, Dragoo J, Eliav E, Benoliel R (2012). Comparison of current perception threshold electrical testing to clinical sensory testing for lingual nerve injuries. J Oral Maxillofac Surg.

[18] Uddin Z, MacDermid JC, Galea V, Gross AR, Pierrynowski MR (2014). The current perception threshold test differentiates categories of mechanical neck disorder. J Orthop Sports Phys Ther.

[19] Cho YW, Kang MS, Kim KT, Do SY, Lim JG, Lee SY, Motamedi GK (2017). Quantitative sensory test for primary restless legs syndrome/Willis–Ekbom disease using the current perception threshold test. Sleep Med.

[20] Chang W, Xu W, Hu R, An Y (2020). Corrigendum: current perception threshold testing in pharyngeal paresthesia patients with depression or anxiety (Neuropsychiatr dis treat. 2020, 16, 1023–1029). Neuropsychiatr Dis Treat.

[21] Nakatani-Enomoto S, Yamazaki M, Kamimura Y, Abe M, Asano K, Enomoto H, Wake K, Watanabe S, Ugawa Y (2019). Frequency-dependent current perception threshold in healthy Japanese adults. Bioelectromagnetics..

[22] Ichiro SS, Shimazu H, Kogure E, Watanabe A, Kobayashi H (2019). Factors affecting and adjustments for sex differences in current perception threshold with transcutaneous electrical stimulation in healthy subjects. Neuromodulation..

[23] Pitei DL, Watkins PJ, Stevens MJ, Edmonds ME (1994). The value of the Neurometer in assessing diabetic neuropathy. Diabet Med.

[24] Gribble PA, Delahunt E, Bleakley CM, Caulfield B, Docherty CL, Fong DTP, Fourchet F, Hertel J, Hiller CE, Kaminski TW, McKeon PO, Refshauge KM, van der Wees P, Vicenzino W, Wikstrom EA (2014). Selection criteria for patients with chronic ankle instability in controlled research: a position statement of the international ankle consortium. J Athl Train.

[25] Boyle KL, Witt P, Riegger-Krugh C. Intrarater and Interrater reliability of the Beighton and Horan joint mobility index. J Athl Train. 2003. PMC314385 14737208

[26] Richardson JTE (2011). Eta squared and partial eta squared as measures of effect size in educational research. Educ Res Rev.

[27] Doherty C, Bleakley C, Hertel J, Caulfield B, Ryan J, Delahunt E (2014). Balance failure in single limb stance due to ankle sprain injury: An analysis of center of pressure using the fractal dimension method. Gait Posture..

[28] Renton T, Thexton A, Hankins M, McGurk M (2003). Quantitative thermosensory testing of the lingual and inferior alveolar nerves in health and after iatrogenic injury. Br J Oral Maxillofac Surg.

[29] Leone C, Truini A (2019). The CPM effect: functional assessment of the diffuse noxious inhibitory control in humans. J Clin Neurophysiol.

[30] Griffioen MA, Greenspan JD, Johantgen M, Von Rueden K, O’Toole RV, Dorsey SG (2018). Quantitative sensory testing and current perception threshold testing in patients with chronic pain following lower extremity fracture. Biol Res Nurs.

[31] Liao MF, Lee M, Hsieh MJ, Cheng MY, Der Lee J, Weng HH (2010). Evaluation of the pathophysiology of classical trigeminal neuralgia by blink reflex study and current perception threshold testing. J Headache Pain.

[32] Sousa ASP, Leite J, Costa B, Santos R (2017). Bilateral proprioceptive evaluation in individuals with unilateral chronic ankle instability. J Athl Train.

[33] Terada M, Bowker S, Thomas AC, Pietrosimone B, Hiller CE, Gribble PA (2016). Corticospinal excitability and inhibition of the soleus in individuals with chronic ankle instability. PM R.

[34] Rosen AB, Yentes JM, McGrath ML, Maerlender AC, Myers SA, Mukherjee M (2019). Alterations in cortical activation among individuals with chronic ankle instability during single-limb postural control. J Athl Train.

[35] Kim K-M, Kim J-S, Cruz-Díaz D, Ryu S, Kang M, Taube W (2019). Changes in spinal and Corticospinal excitability in patients with chronic ankle instability: a systematic review with meta-analysis. J Clin Med.

[36] Kleinrensink GJ, Stoeckart R, Meulstee J, Sukul DMKSK, Vleeming A, Snijders CJ (1994). Lowered motor conduction velocity of the peroneal nerve after inversion trauma. Med Sci Sports Exerc.

[37] Cione JA, Cozzarelli J (2009). Neurometer diagnostic sensory evaluation. Foot Ankle Spec.

